# MiR-146b-5p/TRAF6 axis is essential for *Ginkgo biloba L. extract* GBE to attenuate LPS-induced neuroinflammation

**DOI:** 10.3389/fphar.2022.978587

**Published:** 2022-08-24

**Authors:** Min Liu, Yulin Peng, Yilin Che, Meirong Zhou, Ying Bai, Wei Tang, Shanshan Huang, Baojing Zhang, Sa Deng, Chao Wang, Zhenlong Yu

**Affiliations:** ^1^ Neurology Department, Dalian University Affiliated Xinhua Hospital, Dalian, China; ^2^ College of Pharmacy, Dalian Medical University, Dalian, China; ^3^ The 1st Department of Thoracic Medical Oncology, Second Affiliated Hospital, Dalian Medical University, Dalian, China

**Keywords:** TRAF6, miR-146b-5p, Neuroinflammation, NF-kappa B, GBE

## Abstract

**Background:** Neuroinflammation plays a crucial role in the pathogenesis and progression of various neurodegenerative diseases, including Alzheimer’s disease. The Ginkgo biloba leaf extract (GBE) has been widely used to treat cerebral and peripheral blood circulation disorders. However, its potential targets and underlying mechanisms regarding neuroinflammation have not yet been characterized.

**Aims:** The purpose of this study was to investigate and validate the anti-neuroinflammatory properties of GBE against lipopolysaccharide (LPS)-mediated inflammation and to determine the underlying molecular mechanisms.

**Methods:** The effect of GBE on LPS-induced release of inflammatory cytokines was examined using ELISA and western blot assay. The effects of GBE on NF-κB binding activity and translocation were determined *via* luciferase, streptavidin-agarose pulldown, and immunofluorescence assays. The potential targets of GBE were screened from the GEO and microRNA databases and further identified *via* qPCR, luciferase, gene mutation, and western blot assays.

**Results:** GBE significantly inhibited LPS-induced pro-inflammatory responses in BV-2 and U87 cells, with no obvious cytotoxicity. GBE significantly induced miR-146b-5p expression, which negatively regulated TRAF6 expression by targeting its 3′-UTR. Thus, due to TRAF6 suppression, GBE decreases the transcriptional activity of NF-κB and the expression of pro-inflammatory cytokines, such as interleukin (IL)-1β, IL-6, tumor necrosis factor (TNF)-α, and cyclooxygenase (COX)-2, and finally reverses LPS-induced neuroinflammation.

**Conclusion:** Our study revealed the anti-neuroinflammatory mechanism of GBE through the miR-146b-5p/TRAF6 axis and provided a theoretical basis for its rational clinical application.

## Introduction

Neuroinflammation is a protective mechanism in which inflammatory mediators repair damaged neurons and glial cells during acute neuroinflammation (Cobourne-Duval et al., 2018; von Bernhardi et al., 2015; Yang et al., 2020). However, chronic neuroinflammation often leads to neuronal damage and eventual degeneration (Kempuraj et al., 2016; Cobourne-Duval et al., 2018; Kang et al., 2020). For many years, neuroinflammation has been considered a vital driver of many brain diseases (Bidstrup, 1972; Heneka et al., 2015), such as the neurodegenerative Alzheimer’s disease (AD). Previous studies have suggested that chronic neuroinflammation plays an active role in the development and progression of AD (Zhang et al., 2013; Trovato Salinaro et al., 2018). Therefore, inhibition of chronic neuroinflammation represents an effective therapeutic strategy for neuroinflammation-related and neurodegenerative diseases.

The human brain contains microglia, astrocytes, and neurons. Microglia, like macrophages, are involved in the innate immune response of the central nervous system (CNS) and play a vital role in normal brain development and responses to neuroinflammation (Neumann et al., 2009; Goldmann and Prinz, 2013; Yin et al., 2017). Its abnormal activation notably promotes neuroinflammatory and neurotoxic responses through the release of various pro-inflammatory cytokines and mediators, such as IL-1β, IL-6, TNF-α, COX-2, and iNOS (Block and Hong, 2005; Stansley et al., 2012; Xu et al., 2016; Voet et al., 2019). In addition, activated microglia have been suggested to play a pivotal role in the fate of astrocytes becoming neurotoxic, and impairment of astrocyte function contributes to AD pathophysiology (Hashioka et al., 2021).

TRAF6 (TNF receptor associated factor 6) is a member of the TNF receptor associated factor (TRAF) protein family, which is essential for the regulation of toll like receptor (TLR)-mediated signaling and participates in the inflammatory response (Lv et al., 2018; Min et al., 2018). TRAF6 is an E3 ligase and auto-ubiquitinated through K63-linked ubiquitin chains (Kawai and Akira, 2006), and then activates a complex containing TAK1, TAB2 and TAB3 (Ninomiya-Tsuji et al., 1999; Takaesu et al., 2000; Qian et al., 2001). This complex then induces phosphorylation of the IKK (inhibitor of IκB kinase) complex, leading to activation of NF-κB signaling pathway and induction of expression of genes encoding inflammatory cytokines and mediators, including IL-1β, IL-6, TNF-α, and COX-2. Thus, suppression of TRAF6 expression is an effective strategy for treating inflammatory diseases.

Recently, numerous studies have been conducted that focused on the role of microRNAs (miRNAs) in neuroinflammation and neurodegeneration (Lopez-Ramirez et al., 2016; Konovalova et al., 2019). MiRNAs are small non-coding RNAs that can regulate post-transcriptional gene expression by binding to regions of partial complementarity in the 3′-untranslated region (UTR) of messenger RNA (mRNA) to induce mRNA degradation and/or inhibit translation (Bartel, 2009). They are particularly abundant within neurons and involved in the post-mitotic survival of neurons and neuroprotection (Boudreau et al., 2014). Indeed, knockout of miRNAs has been shown to lead to neurodegeneration in many animal models. In addition, evidence from human tissues demonstrates that malregulated miRNA expression performs important functions in the development of neurodegenerative diseases (Reddy et al., 2017). Based on these findings, it is reasonable to hypothesize that miRNAs could be the targets of future therapeutic development for neurodegenerative diseases.

Owing to their potential pharmacological activities with low toxicity, natural products have attracted significant attention from researchers. The *Ginkgo biloba L.* leaf extraction (GBE) has a long history of use in traditional Chinese medicine with broad applications in improving memory and cerebrovascular diseases (Li et al., 2017). GBE has been shown to possess various physiological and pharmacological properties, such as antibacterial l (Hu et al., 2019), DNA protective activities (Li et al., 2019) and mitochondrial function recovery (Baliutyte et al., 2014). These biological functions are mainly associated with its anti-inflammatory (Kotakadi et al., 2008) and antioxidant activities (Singh et al., 2019). Recent studies have suggested the therapeutic activities of GBE for the treatment of AD, cardiovascular disease, dementia, memory loss, and cerebral ischemia (Ihl, 2013; Strohle et al., 2015). In clinical practice, GBE has been used as an injection for treatment of cerebral and peripheral blood circulation disorders, such as acute and chronic cerebral insufficiency and their sequelae. However, the potential targets and underlying mechanisms of GBE have not yet been characterized, which severely limits its clinical application in other diseases, such as neuroinflammatory related neurodegenerative AD. Therefore, it is necessary to explore the mechanism of GBE action to broaden its clinical application.

To our best knowledge, it is the first time to unequivocally identify miR-146b-5p as an anti-inflammatory target of GBE using a combination of molecular pharmacological phenotyping and bioinformatics analysis. Such anti-inflammatory effects of GBE were mediated by miR-146b-5p/TRAF6 axis and NF-κB signaling. Our results thus reveal the anti-inflammatory properties of GBE, and provide an advanced understanding of miR-146b-5p function.

## Materials and methods

### Reagents and antibodies

*Ginkgo biloba L.* leaf extract injection (GBE) was purchased from Youcare Pharmaceutical Group (Beijing, China, Approval No. H20070226, Supporting Information 1). Its initial concentration is 3.5 mg/ml and prepared in aqueous solvent with excipients such as sorbitol, ethanol, sodium hydroxide. Lipopolysaccharide (LPS, #L2880) were obtained from sigma, and prepared in DMEM medium at concentration of 10 mg/ml. Primary antibodies for COX-2 (#4842, 1:1000), IκB-α (L35A5) (#4814, 1:1000), p-IκB-α (14D4) (#2859, 1:1000), NF-κB p65 (D14E12) (#8242, 1:1000) and p-p65 (93H1) (#3033, 1:1000), NF-κB p50 (D4P4D) (#13586, 1:1000) were obtained from Cell Signaling Technology, Inc. (Danvers, MA, United States). Primary antibodies for TNF-α (7B8A11) (#60291-1-Ig, 1:1000), IL-6 (#21865-1-AP, 1:1000), GAPDH (1E6D9) (#60004-1-Ig, 1:1000), β-actin (2D4H5) (#66009-1-Ig, 1:1000) and Lamin B1 (#12987-1-AP, 1:1000) were obtained from Proteintech Group Inc. (Rosemount, IL, United States). Dulbecco’s modified Eagle’s medium (DMEM) and fetal bovine serum (FBS) were obtained from Gibco Laboratories.

### Cell culture and treatment

Immortalized mouse BV-2 microglial cell line and human U87 glioblastoma cells were cultured in DMEM medium with 10% FBS and 1% penicillin/streptomycin at 37 °C in a humidified atmosphere containing 95% air and 5% CO_2_, and the medium was changed every 2 days. The cells were split with 0.25% trypsin when they reached 90% confluence and sub-cultured for further passages. Cells were pretreated with various concentrations of GBE for 1 h, and treated with or without LPS (0.1 µg/ml) for 24 h.

### Cell viability assay

Cell viability was determined using CCK-8 (Cell Counting Kit-8) assay. In brief, 5 × 10³ BV-2 or U87 cells were inoculated on 96-well to adhere overnight. Then, the cells were incubated with a fresh medium containing GBE (5–100 µg/ml) for 1 h, followed by lipopolysaccharide (LPS) (0.1 µg/ml) treatment. After treatment for 24 h, CCK-8 solution (10 μl) was added, and the absorbance was measured at 450 nm using a Biotek Multimode Plate Reader. Cell viability in the vehicle control group was considered to be 100%. Each assay was performed in triplicates.

### Determination of the release of tumor necrosis factor-α, interleukin-1β, and interleukin-6

BV-2 or U87 cells were pre-incubated with various concentrations of GBE for 1 h, followed by LPS (0.1 µg/ml) treatment. After incubation for another 24 h, supernatants were collected and centrifuged at 1500 × g for 10 min at 4°C. TNF-α, IL-1β, and IL-6 productions were assessed in the supernatants using ELISA kits (Elabscience Biotechnology Co., Ltd., Wuhan, China), according to the manufacturer’s instructions.

### Western blot analysis

Protein (30–50 μg) lysates were separated *via* SDS-PAGE using 10–12% gel, and then transferred to a PVDF membrane. After blocking with 5% nonfat milk, the PVDF membrane was washed and probed with specific primary and secondary antibodies. The protein bands were visualized by enhanced chemiluminescence. The experiments were performed at least thrice.

### Dual-luciferase reporter assay

293T cells in a six-well plate were transfected with pNFκB-luc (Beyotime Biotechnology) plasmid, TRAF6 wild-type, or TRAF6 mutated 3′-UTR, with Lipofectamine 2000 Reagent (Invitrogen, Grand Island, NY, United States). After transfection, the cells were pre-treated with GBE (25 and 50 µg/ml) for 1 h, followed by LPS (0.1 μg/ml) treatment for 24 h. The luciferase activity was measure using a dual-luciferase reporter assay system (Promega, Madison, WI, United States), and normalized to the corresponding Renilla luciferase activity.

### Cell transfection assay

MiR-146b-5p mimic (miR-146b) and its control (miR-ctrl) and miR-146b-5p inhibitor (antimiR-146b) and its control (antimiR-ctrl) were obtained from General Biological System (Anhui, China). Transfection was performed using Lipofectamine 2000 when the confluence of BV-2 or U87 cells reached approximately 70%. After transfection for 24 h, the cells were used for further study.

### Detection of DNA/protein binding *via* streptavidin-agarose pulldown assay

A streptavidin-agarose pulldown assay was performed to detect the binding activity of NF-κB p65/p50 to the COX-2 core promoter probe (−20 nt to −498 nt), which was biotin-labeled. Briefly, 400 μg of nuclear extract proteins, 4 μg of biotinylated DNA probe, and 40 μl of 4% streptavidin-conjugated agarose beads were mixed with appropriate volume of PBSi buffer (PBS buffer with 1 mM EDTA, 1 mM DTT, and protease inhibitor cocktail complete) at room temperature (25°C) for 5 h on a rotating shaker, with a total volume of 400 μl. After washing with PBSi buffer twice, the beads were resuspended in 2X SDS-PAGE loading buffer and boiled for 10 min. The supernatant was analyzed by western blotting.

### Confocal immunofluorescence

Immunofluorescence staining was performed in BV-2 cells that were cultured in chamber slides with the indicated GBE and/or LPS treatment. After fixture and permeabilization with 4% paraformaldehyde and 0.2% TritonX-100, the slides were probed with primary against p65 and p50 overnight at 4 °C. Following washing with PBS, samples were incubated with fluorescein isothiocyanate- (#SA00003-1, 1:50) and rhodamine-(# SA00007-2, 1:50) conjugated secondary antibodies for 1 h. Then, the slides were stained with DAPI to counterstain the cell nuclei. The samples were examined under a Leica DM14000B confocal microscope (Leica, Germany).

### Statistical analysis

SPSS 17.0 (IBM Corp., Armonk, NY, United States) was used to perform all the statistical analyses. Unpaired Student’s t-test was used to assess differences between two groups, whereas analysis of variance (ANOVA) followed by the least significant difference test was used for comparisons of multiple groups. Differences were considered statistically significant at *p* < 0.05.

## Results

### Ginkgo biloba leaf extract inhibited lipopolysaccharide-induced pro-inflammatory response in BV-2 and U87 cells

**FIGURE 1:**
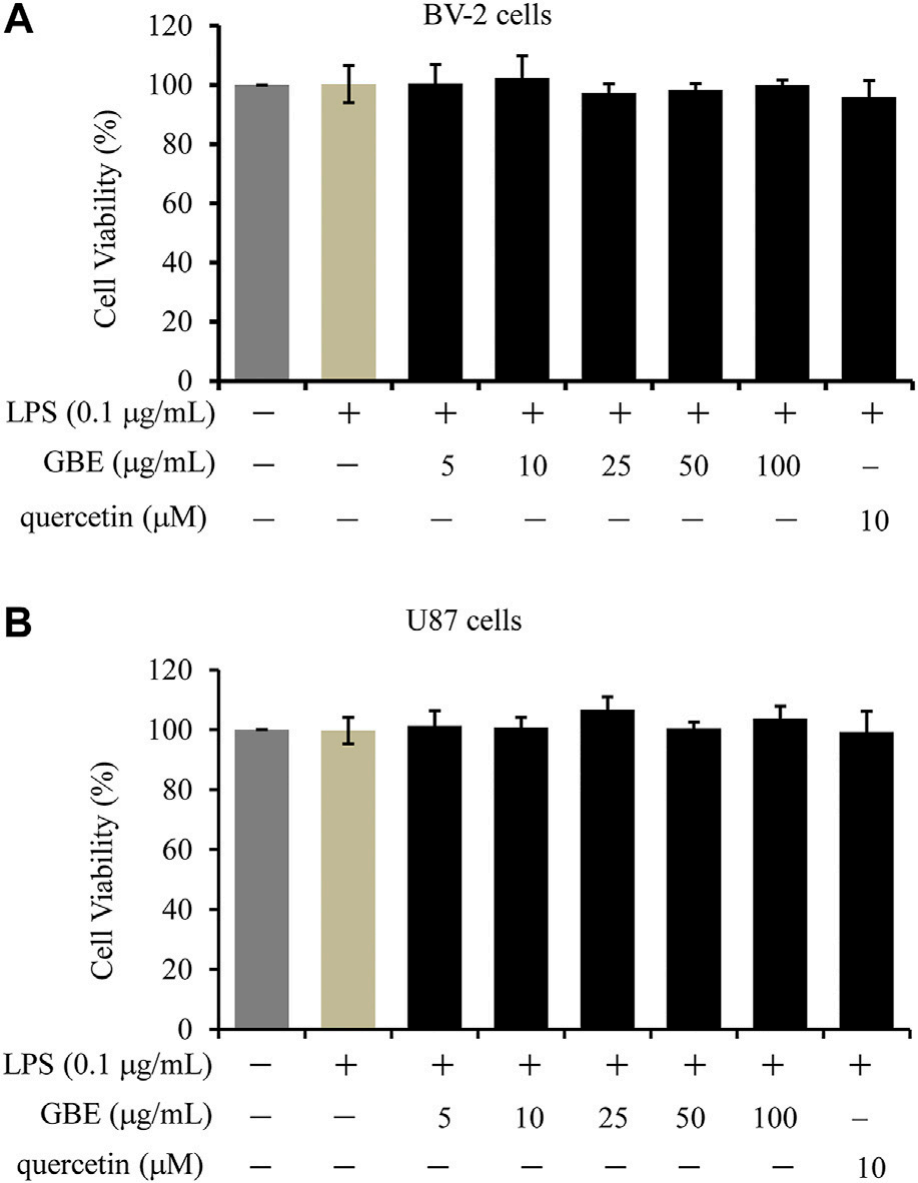
The effects of GBE on the viability of BV-2 microglial cells and U87 cells. BV-2 microglial cells **(A)** and U87 cells **(B)** were treated with GBE (5–100 µg/ml) or quercetin (10 µM) for 25 h with LPS treatment for the last 24 h. The solvent controls of GBE, LPS and quercetin were physiological saline solution, DMEM medium and DMSO, respectively. Cell viability was measured by CCK-8 assay, and data were normalized as 100% of control. Statistical analyses were performed using one-way ANOVA. The data are presented as the mean ± SD of three independent experiments.

To investigate the cytotoxic effects of GBE on cell viability, a CCK-8 assay was performed on BV-2 microglial cells. LPS (0.1 μg/ml) had no obvious effects on the viability of BV-2 cells, and the combination with various GBE concentrations up to 100 µg/ml did not change cell viability compared to vehicle only (DMSO)-treated cells (Figure 1A). Similar results were observed for U87 cells (Figure 1B). Therefore, we used doses of 25 and 50 µg/ml GBE in all subsequent experiments.

**FIGURE 2:**
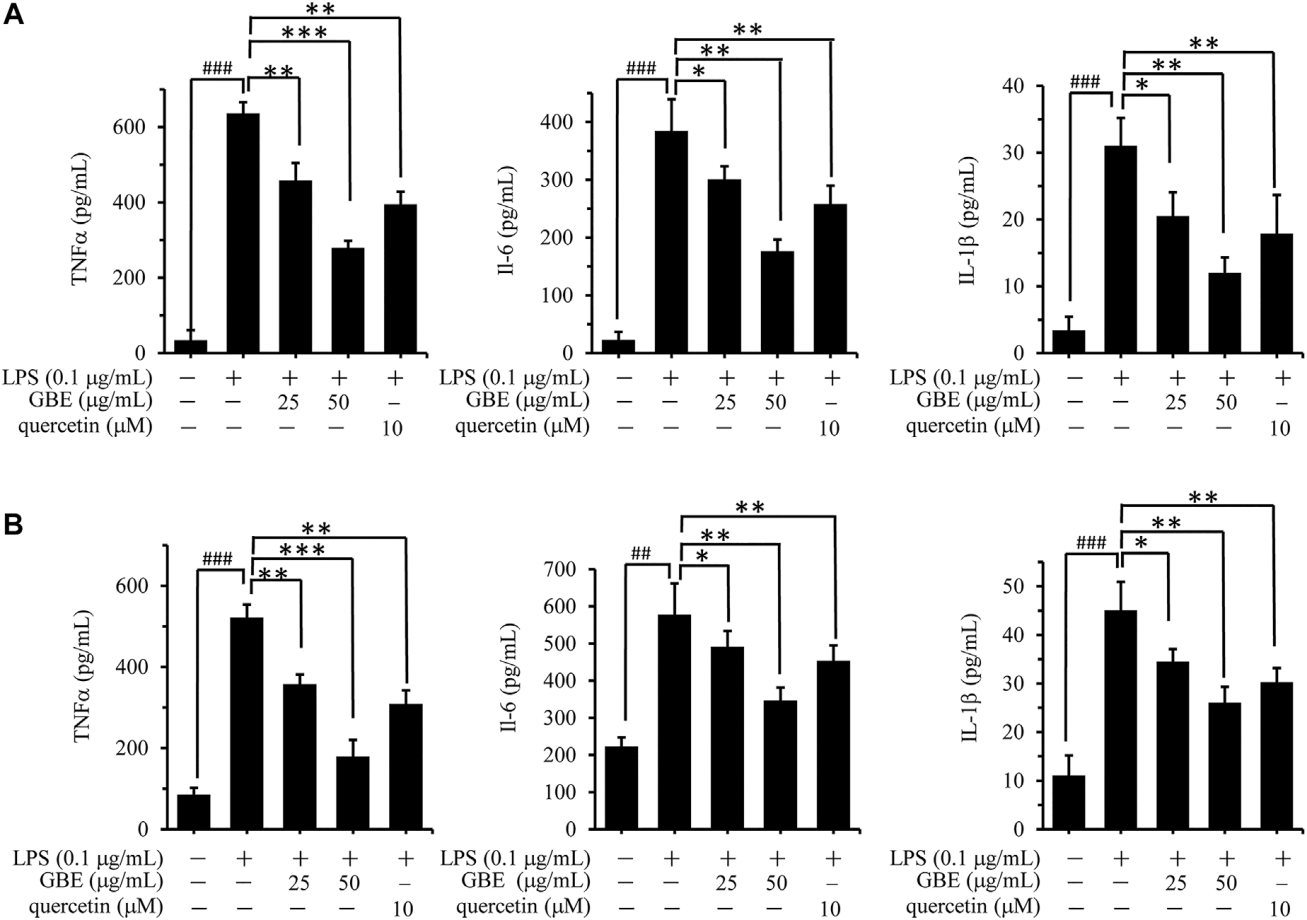
Effects of GBE on the release of TNF-α, IL-1β, and IL-6 in lipopolysaccharide (LPS)-activated BV-2 microglia cells and U87 cells. BV-2 microglial cells **(A)** and U87 cells **(B)** were pre-treated with GBE (25 and 50 µg/ml) or quercetin (10 µM) for 1 h, and then incubated with or without LPS (0.1 µg/ml) for 24 h. After incubation, cell supernatants were collected and release of TNF-α, IL-6, and IL-1β was measured using ELISA kit. The data are presented as the mean ± SD of three independent experiments. (*p < 0.05, **p < 0.01, ***p < 0.001, GBE treatment vs. LPS-stimulated positive control; ### *p* < 0.001, LPS-stimulated positive control vs. vehicle control groups).

LPS stimulation can activate microglial cells, accompanied by the release of various inflammatory cytokines, including TNF-α, IL-1β, and IL-6 (Mar et al., 2015; Guo et al., 2016). To evaluate the inhibitory effect of GBE on LPS-induced release of these cytokines, BV-2 cells were pre-incubated with GBE (0, 25, and 50 µg/ml) for 1 h and then stimulated with LPS (0.1 μg/ml) for 24 h. LPS stimulation increased the robust amounts of TNFα, IL-1β, and IL-6 compared to the vehicle control, and this increase was significantly inhibited by GBE in a dose-dependent manner in BV-2 cells (Figure 2A). Similar inhibitory effects were also observed in LPS-induced U87 cells (Figure 2B).

Additionally, quercetin was selected as a positive control drug (Tang et al., 2019), and its cytotoxicity and anti-inflammatory effects were also evaluated. The results showed that quercetin has no obvious effects on the cell viability of both BV-2 and U87 cells, however, it could effectively weaken LPS-induced release of TNFα, IL-1β or IL-6 (Figures 1, 2).

### Ginkgo biloba leaf extract suppressed the expression of lipopolysaccharide-stimulated inflammatory cytokines and activation of the NF-κB pathway

**FIGURE 3:**
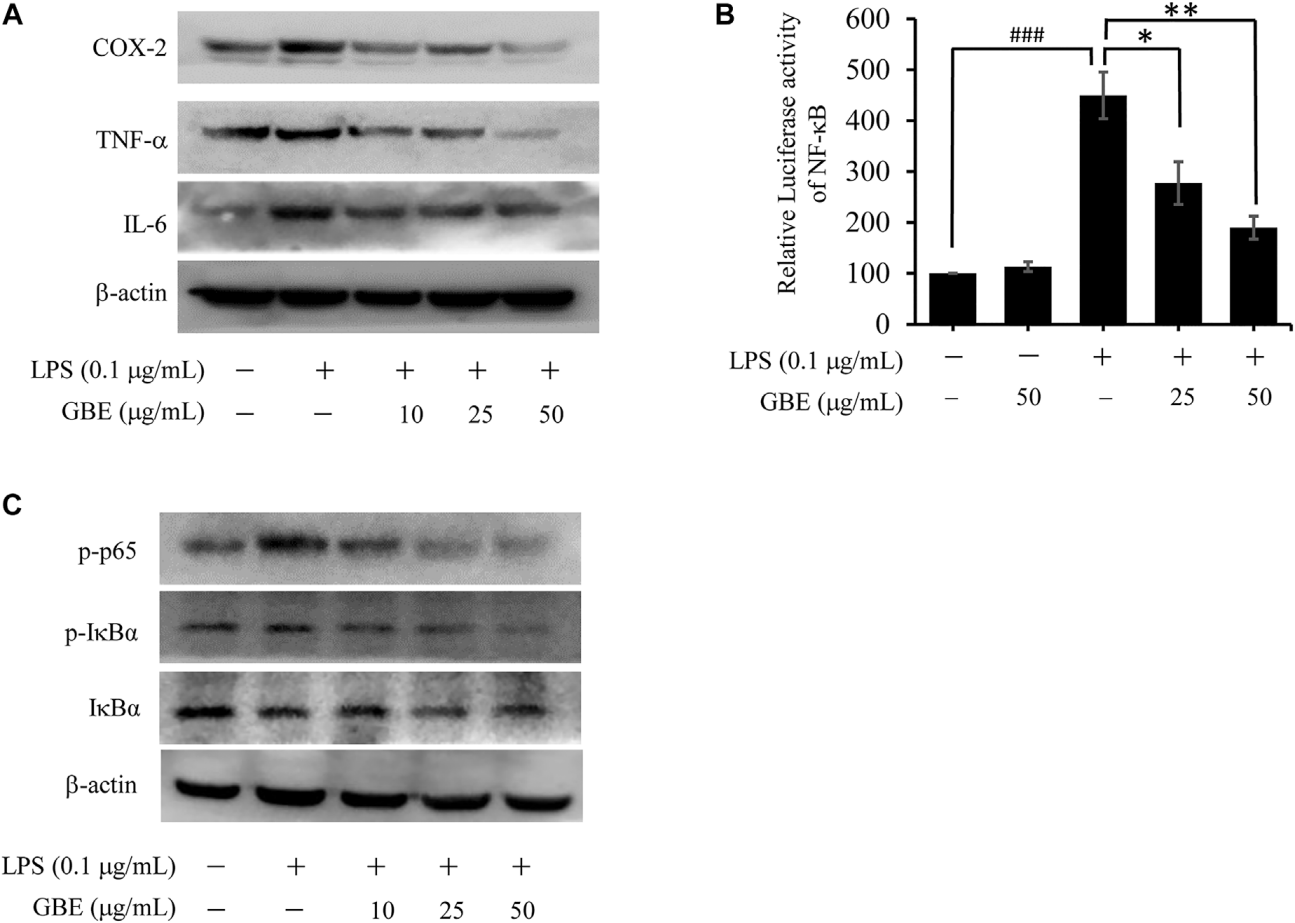
Effects of GBE on the expression of inflammatory cytokines and NF-κB pathway in LPS-stimulated BV-2 cells. BV-2 cells were pre-treated with GBE (25 and 50 µg/ml) for 1 h, and then incubated with or without LPS (0.1 µg/ml) for 24 h. **(A)** The expression of TNF-α, IL-6, and COX-2 was analyzed using western blotting in BV-2 cells. **(B)** 293T cells were cotransfected with pNFκB-luc with Renilla luciferase reporter (as internal control) for 24 h, and then pre-treated with GBE (25 and 50 µg/ml) for 1 h; thereafter, the cells were incubated with or without LPS (0.1 µg/ml) for 24 h. Luciferase activity was determined using a dual-luciferase reporter assay system (*p < 0.05, **p < 0.01, GBE treatment vs. LPS-stimulated positive control; ### *p* < 0.001, LPS-stimulated positive control vs. vehicle control groups). **(C)** The expression of p-p65, p-IκBα, and IκBα were analyzed using western blotting in BV-2 cells.

As inflammatory mediators play an important role in chronic inflammation, we examined the effects of GBE on the expression of TNF-α, IL-6, and COX-2 in LPS-stimulated BV-2 cells. The results revealed that GBE treatment significantly suppressed LPS-mediated expression of TNF-α, IL-6, and COX-2 (Figure 3A). Because the expression of these three major inflammatory cytokines is regulated by the transcription factor NF-κB (Zeinali et al., 2017), we examined the effect of GBE on NF-κB activity by a dual-luciferase reporter assay. As shown in Figure 3B, when treated with LPS, luciferase activity was immediately enhanced, and the high activity decreased in a dose-dependent manner with GBE treatment. Furthermore, as NF-κB activation depends on phosphorylation of IκB proteins, we evaluated the effects of GBE on phosphorylation of IκBα and p65. As expected, GBE clearly suppressed LPS-mediated phosphorylation of IκBα and p65 (Figure 3C).

### Ginkgo biloba leaf extract blocked NF-κB binding activity and translocation

**FIGURE 4:**
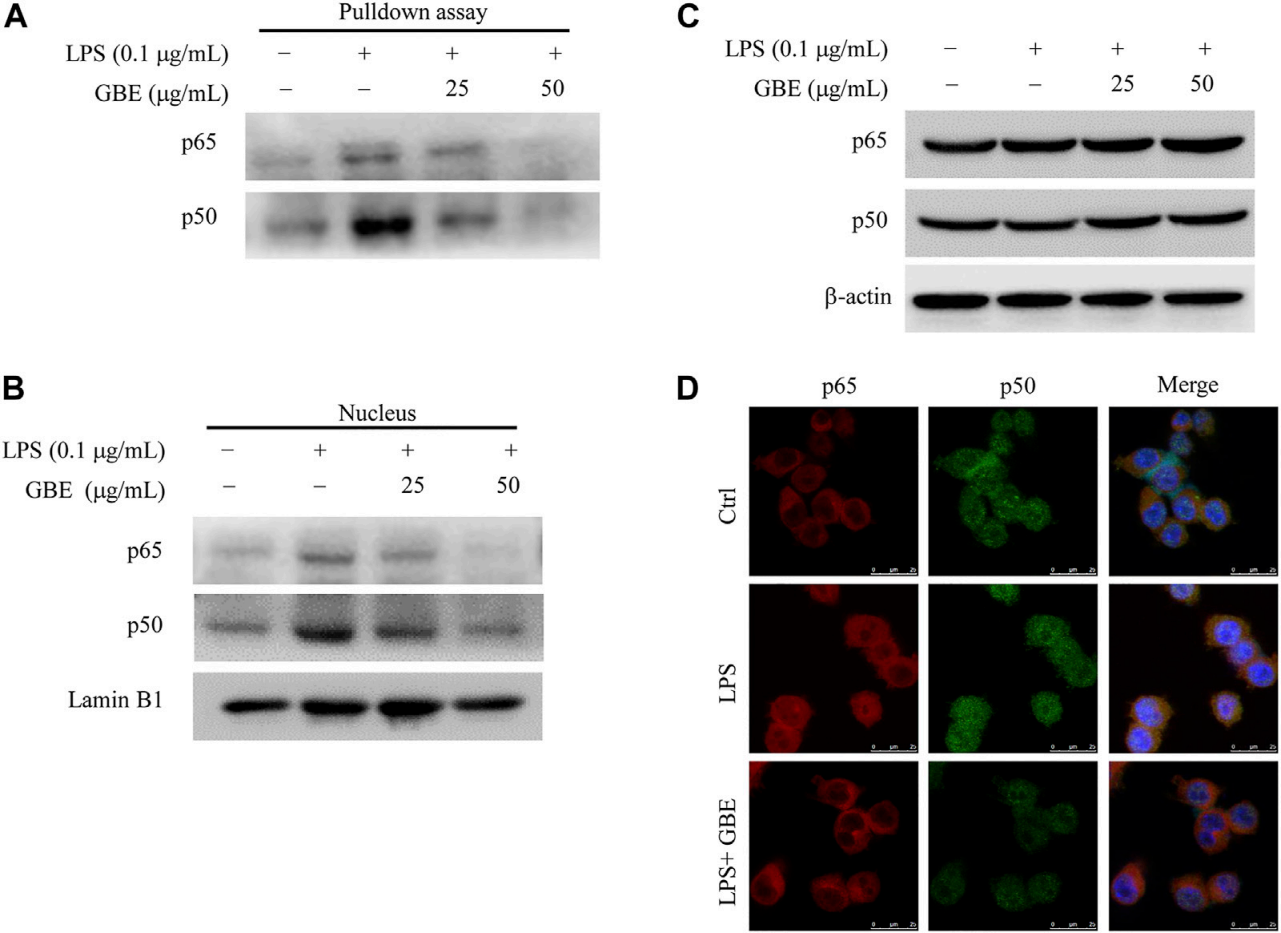
Effects of GBE on NF-κB binding activity and translocation. BV-2 cells were pre-treated with GBE (25 and 50 µg/ml) for 1 h, and then incubated with or without LPS (0.1 µg/ml) for 24 h. **(A)** The binding of NF-κB p65 and p50 to COX-2 promoter was analyzed using streptavidin-agarose pulldown assay. The protein levels of NF-κB p65 and p50 in whole-cell lysates **(B)** and nucleus **(C)** were measured by western blotting analysis. β-actin and Lamin B1 were used as controls for sample loading. **(D)** The translocation of p50 and p65 from cytoplasm to nucleus were observed in BV-2 cells with indicated treatment by immunofluorescence imaging analysis. Confocal microscopy analysis showed that LPS could effectively promote p50/p65 entry into the nucleus, while GBE treatment significantly reduced their entry into the nucleus.

We further assessed the effect of GBE on the binding activity of NF-κB to the COX-2 promoter using a streptavidin-agarose pulldown assay. As shown in Figure 4A, GBE treatment markedly attenuated the binding of NF-κB p50/p65 subunits to the COX-2 promoter DNA probe (Figure 4A) compared with LPS treatment. Moreover, we detected whether GBE suppressed the expression of NF-κB p50/p65 subunits. The results showed that GBE significantly decreased p65/p50 protein levels in nuclear lysates (Figure 4B), but had no obvious effects on their protein expression in LPS-stimulated BV-2 cells (Figure 4C). Based on the above results, we hypothesized that GBE markedly inhibits the translocation of NF-κB p65/p50 subunits from the cell cytoplasm to the nucleus. We next performed the immunofluorescence assay to verify this hypothesis. As expected, GBE markedly inhibited the translocation of NF-κB p65/p50 subunits in BV-2 cells (Figure 4D). The above results indicate that the anti-neuroinflammatory effects of GBE might be mediated by the inhibition of NF-κB translocation from the cytoplasm to the nucleus, which then further inhibits COX-2 expression.

### MiR-146b-5p was identified as a potential target of ginkgo biloba leaf extract

**FIGURE 5:**
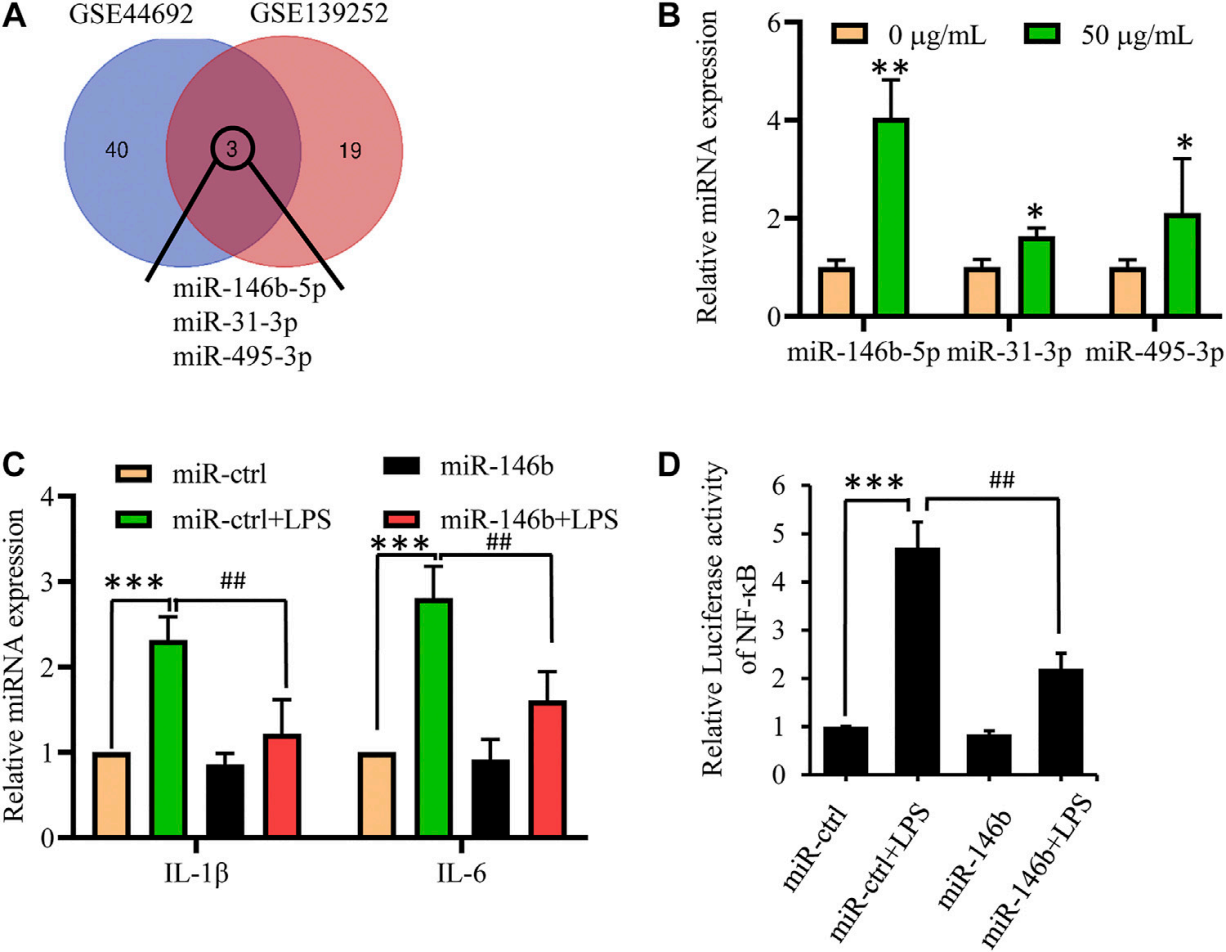
MiR-146b-5p is a potential target of GBE to suppress inflammation response. **(A)** Gene expression omnibus (GEO) database (GSE44692, GSE139252) was screened for differentially expressed miRNAs for both GBE and Alzheimer’s disease. **(B)** U87 cells were treated with GBE (0 and 50 µg/ml) for 24 h, and the expression of miR-146b-5p, miR-31-3p, and miR-495-3p were detected by qPCR (*p < 0.05, **p < 0.01 vs. negative control group). **(C)** U87 cells were transfected with miR-146b-5p mimics for 24 h, and then incubated with or without LPS (0.1 µg/ml) for 24 h. The expressions of IL-6 and IL-1β were measured by qPCR. **(D)** 293T cells were cotransfected with pNFκB-luc, renilla luciferase reporter, and miR-146b-5p mimic for 24 h; thereafter, cells were incubated with or without LPS (0.1 µg/ml) for 24 h. Luciferase activity was determined using a dual-luciferase reporter assay system (**p < 0.01, LPS-stimulated positive control vs. vehicle control groups; ##*p* < 0.01, miR-146b-5p treatment vs. LPS-stimulated positive control).

To further investigate the mechanism of suppression of neuroinflammation progression by GBE, we focused on microRNAs that play a vital role in the development of neuroinflammation. After screening differentially expressed miRNAs for both GBE and AD in the gene expression omnibus (GEO) database (GSE44692 and GSE139252) (*p* < 0.05, log|FC| ≥ 2), we found that miR-146b-5p, miR-31-3p, and miR-495-3p were present in both GBE-treated and AD groups (Figure 5A). Quantitative PCR (qPCR) results showed that miR-146b-5p expression changed significantly in GBE-treated cells (Figure 5B). Therefore, hsa-miR-146b-5p was selected as a potential target gene of GBE for further studies. As shown in Figure 5C, miR-146b-5p overexpression significantly reduced the LPS-induced release of IL-1β and IL-6 but had a minor effect on endogenous expression. Furthermore, luciferase reporter assays also showed that miR-146b-5p overexpression reduced reporter activity driven by the NF-κB promoter (Figure 5D). These results suggest that miR-146b-5p suppresses the inflammatory response.

**FIGURE 6:**
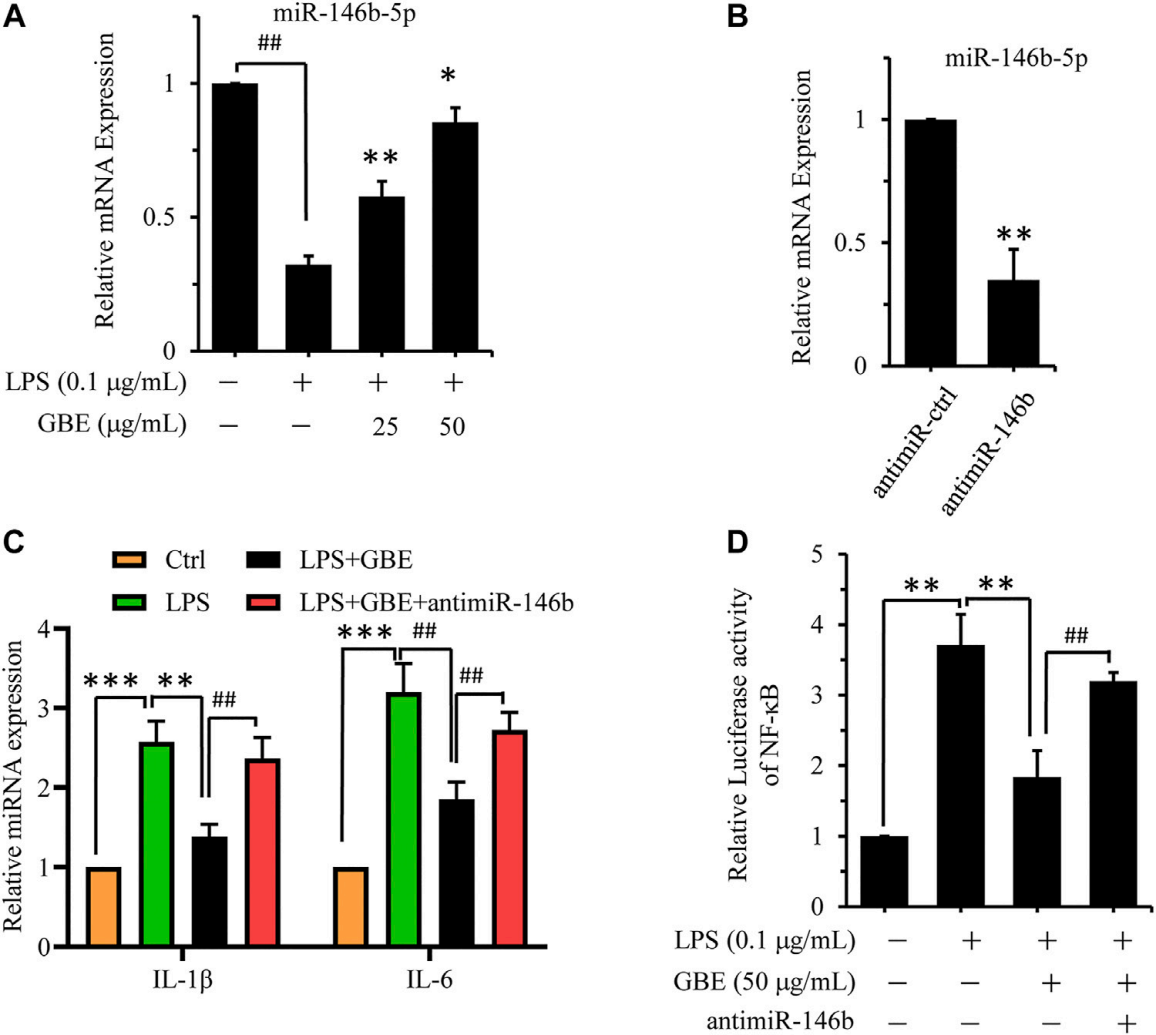
GBE suppressed neuroinflammation *via* upregulating miR-146b-5p expression. **(A)** U87 cells were pre-treated with GBE (25 and 50 µg/ml) for 1 h, and then incubated with or without LPS (0.1 µg/ml) for 24 h. The expression of miR-146b-5p was detected by qPCR (## *p* < 0.01 vs. vehicle control, *p < 0.05, **p < 0.01 vs. LPS group). **(B)** U87 cells were transfected with miR-146b-5p inhibitor for 24 h, and then the expression of miR-146b-5p was detected by qPCR (**p < 0.01 vs. inhibitor negative control (NC)). **(C)** U87 cells were transfected with miR-146b-5p inhibitors for 24 h, and then pre-treated with GBE (50 µg/ml) for 1 h, followed by incubation with or without LPS (0.1 µg/ml) for 24 h. The expression of IL-6 and IL-1β were measured using qPCR (**p < 0.01 vs. LPS group; ## *p* < 0.001 vs. GBE group). **(D)** 293T cells were cotransfected with pNFκB-luc, renilla luciferase reporter, and miR-146b-5p inhibitors for 24 h; thereafter, cells were pre-treated with GBE (50 µg/ml) for 1 h, followed by incubation with or without LPS (0.1 µg/ml) for 24 h. Luciferase activity was determined using a dual-luciferase reporter assay system (**p < 0.01 vs. LPS group; ## *p* < 0.001 vs. GBE group).

Next, we examined the effect of GBE on miR-146b-5p expression in LPS-stimulated U87 astrocytes using qPCR. From Figure 6A, the results showed that miR-146b-5p expression was downregulated in the LPS group compared to that in the negative control (NC) group. GBE treatment clearly reversed the expression of miR-146b-5p in LPS-simulated U87 astrocytes in a dose-dependent manner. The results indicate that miR-146b-5p may participate in the regulation of GBE-mediated anti-neuroinflammatory activity. To further examine the effect of miR-146b-5p in GBE-treated astrocytes, a miR-146b-5p inhibitor was employed to exogenously regulate miR-146b-5p expression in LPS-stimulated U87 astrocytes. We found that miR-146b-5p expression was clearly reduced by transfection with the miR-146b-5p inhibitor (antimiR-146-5p), compared to cells transfected with the NC inhibitor (antimiR-control) (Figure 6B). MiR-146b-5p suppression reversed the GBE-induced inflammatory cytokine reduction in LPS-stimulated U87 astrocytes (Figure 6C). Furthermore, the luciferase activity, inhibited by GBE, was also attenuated by miR-146b-5p suppression (Figure 6D). The above data indicate that GBE suppresses neuroinflammation by upregulating miR-146b-5p expression.

### Ginkgo biloba leaf extract -induced miR-146b-5p overexpression negatively regulates TNF receptor associated factor 6 expression by targeting its 3′-untranslated region

**FIGURE 7:**
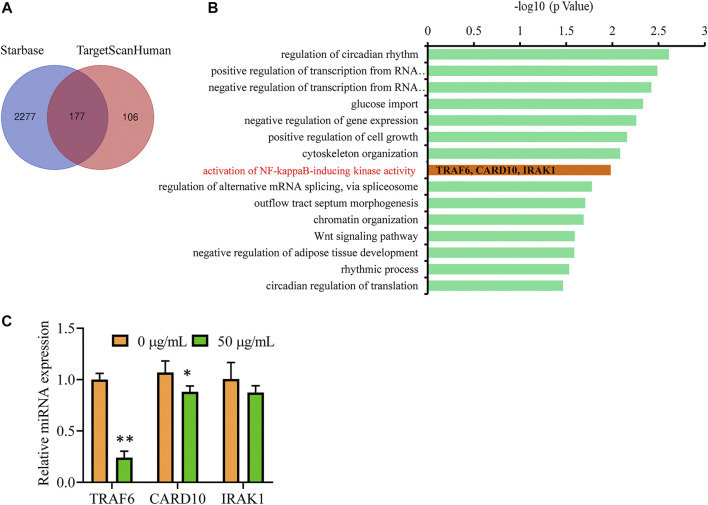
TRAF6 is a potential target gene of miR-146b-5p to suppress inflammation response. **(A)** Venn diagram displaying the numbers of the common target genes of hsa-miR-146b-5p in Starbase and TargetScanHuman databases. **(B)** The gene ontology enrichment of the 177 candidates. The Y-axis lists the name of each category, and the X-axis shows the -log10 values (*p*-value). **(C)** U87 cells were treated with GBE (0 and 50 µg/ml) for 24 h, and the expression of CARD10, IRAK1, and TRAF6 were detected using qPCR. *p < 0.05, **p < 0.01 vs. vehicle control.

To identify the target genes that are regulated by miR-146b-5p, we screened the Starbase and TargetScanHuman databases. In total, 177 differentially expressed genes were identified as candidate targets of miR-146b-5p, as they were observed in both the databases (Figure 7A). After gene ontology analysis of the 177 candidate proteins, an inflammation-related functional cluster of “activation of NF-κB-inducing kinase activity” was identified, mainly including CARD10, IRAK1, and TRAF6 (Figure 7B). This finding was consistent with the experimental results mentioned above. qPCR analysis further revealed that TRAF6, an upstream regulator of NF-κB, showed the greatest change in expression with miR-146b-5p treatment (Figure 7C).

**FIGURE 8:**
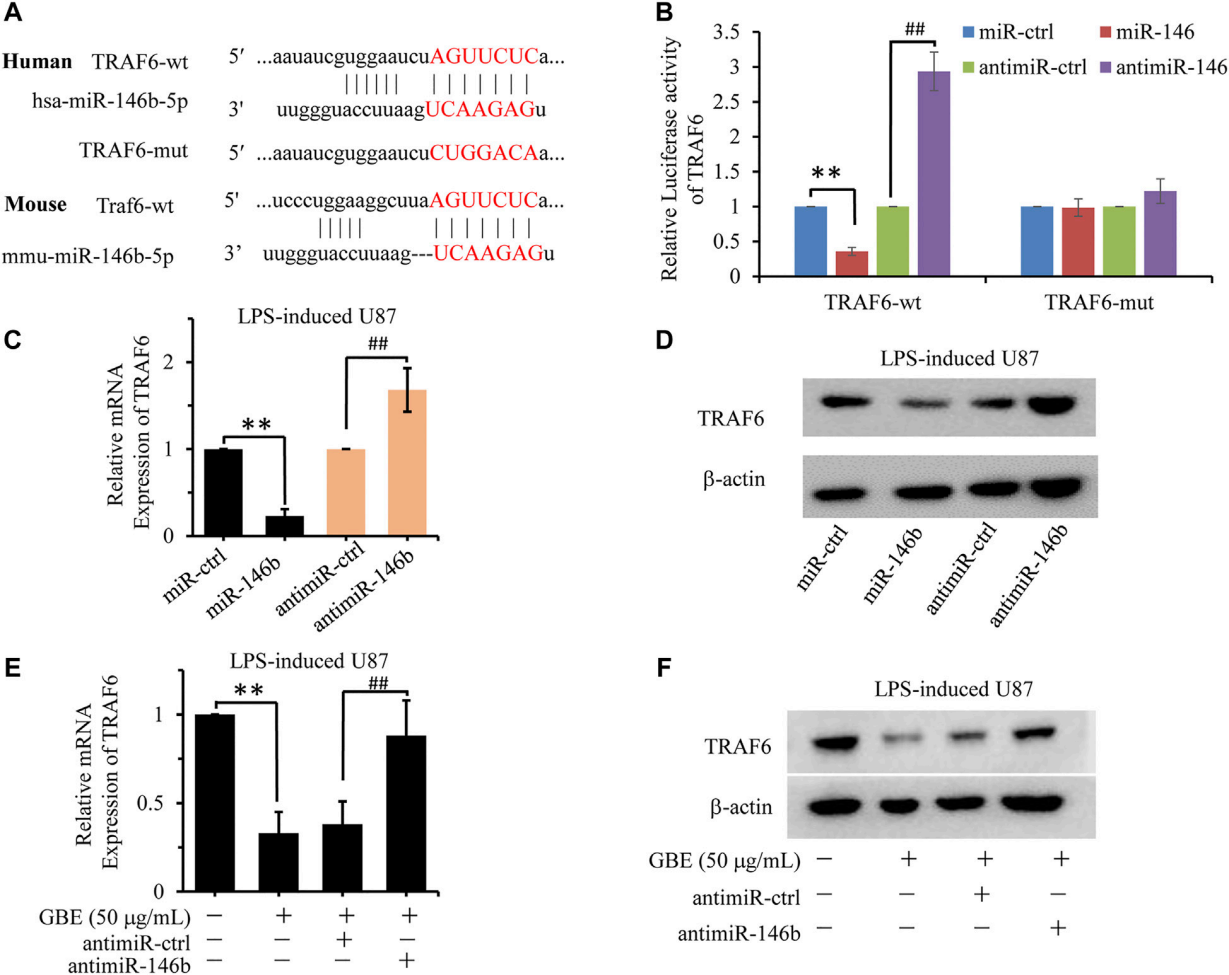
GBE downregulated TRAF6-mediated inflammation response *via* miR-146b-5p. **(A)** The putative target sites of hsa-miR-146b-5p or mmu-miR-146b-5p in TRAF6 were predicted by TargetScanHuman. The mutant binding sequence in TRAF6 with miR-146b-5p is also shown. **(B)** Luciferase assay of 293T cells co-transfected with firefly luciferase constructs containing the TRAF6 wild-type or mutated 3′-UTRs and miR-146b-5p mimic, negative control (NC) mimic, miR-146b-5p inhibitor, or inhibitor NC. **p < 0.01 vs. mimic NC, ## *p* < 0.01 vs. inhibitor NC. **(C,D)** The expression of TRAF6 was detected at both mRNA **(C)** and protein levels **(D)** in LPS-induced U87 cells with miR-146b-5p mimic, NC mimic, miR-146b-5p inhibitor, or inhibitor NC treatment. **p < 0.01 vs. NC mimic, ## *p* < 0.01 vs. inhibitor NC. **(E,F)** The expression of TRAF6 was detected at both mRNA **(E)** and protein levels **(F)** in LPS-induced U87 cells with GBE (0 and 50 µg/ml), miR-146b-5p inhibitor or inhibitor NC treatment. **p < 0.01 vs. vehicle control, ## *p* < 0.01 vs. inhibitor NC.

The complementary sites between miR-146b-5p and TRAF6 predicted by the TargetScanHuman database (Figure 8A) share high homology between mice and humans. Therefore, we investigated the association between miR-146b-5p expression and TRAF6 expression. A dual-luciferase reporter assay was performed to validate whether miR-146b-5p could bind to the 3′-UTR of TRAF6. As shown in Figure 8B, the luciferase activity of TRAF6-wild type (TRAF6-wt) was markedly decreased in the miR-146b-5p group and increased in the anti-miR-146b group compared with the miR-control group. Additionally, the inhibitory effect of miR-146b-5p on TRAF luciferase was abrogated in the mutation group compared to that in the wild-type group. Furthermore, miR-146b-5p overexpression reduced TRAF6 expression at both the mRNA and protein levels in LPS-induced U87 astrocytes compared to that in control cells (Figures 8C,D). In contrast, miR-146b-5p inhibitor stimulation increased the mRNA and protein levels of TRAF6 (Figures 8C,D), confirming that miR-146b-5p negatively regulates the expression of TRAF6. These results support that miR-146b-5p could directly bind to the 3′-UTR of TRAF6 and suppress its expression.

To further investigate the molecular mechanism of GBE function in TRAF6 expression, the effect of different concentrations of GBE on miR-146b-5p expression was evaluated. As shown in Figures 8E,F, GBE inhibited TRAF6 expression at both the mRNA and protein levels in LPS-stimulated U87 astrocytes. This inhibition was reversed by miR-146b-5p suppression, implying that GBE decreases TRAF6 levels in a miR-146b-5p-dependent manner.

## Discussion

Microglial cells are immortal, derived from murine microglia, and share similar properties under inflammatory conditions (Henn et al., 2009). Its abnormal activation promotes the production of pro-inflammatory cytokines and neurotoxic materials, which induce neuroinflammation in microglia (Guo et al., 2016) and subsequently initiate various neurodegenerative diseases (Xie et al., 2013), such as AD. Thus, targeting microglial activation and the release of inflammatory molecules is of significant importance in the treatment of neuroinflammation. In the present study, LPS-induced microglial BV-2 cells and U87 astrocytes were used as *in vitro* models of neuroinflammation to investigate the anti-inflammatory mechanisms of GBE. The major findings revealed that GBE exerted anti-inflammatory effects by upregulating miR-146b-5p expression to inhibit TRAF6 expression, thereby attenuating the NF-κB signaling pathway to inhibit LPS-induced production of pro-inflammatory mediators such as TNFα, IL-6, and IL-1β.

Currently, the clinical application of GBE has been mainly in its positive effects on circulation, such as for the treatment of atherosclerosis, cerebrovascular insufficiency and cerebral stroke. Information from laboratory data showed that GBE has an anti-neuroinflammatory effect (Huang et al., 2021). However, its underlying mechanism still remains unclear. In the present study, we revealed that miR-146b-5p is a novel molecular target for GBE to inhibit neuroinflammation using a combination of molecular pharmacological phenotyping and bioinformatics analysis. To our best knowledge, our study demonstrated for the first time that GBE played an anti-neuroinflammatory role by upregulating the expression of miR-146b-5p, which binds to the 3′UTR of TRAF6 to inhibit its expression, thereby attenuating the NF-κB signaling pathway activation. Our study also provided a theoretical basis of GBE for designing therapeutic strategies, for basic understanding of the underlying neurodegenerative processes, and for a better understanding of its effectiveness and complexity. In addition, our findings provide a drug screening system based on miR-146b-5p/TRAF6 (Figure 8B) that could be used to screen for natural products that inhibited neuroinflammation *via* miR-146b-5p/TRAF6.

Neuroinflammation is a key factor of Alzheimer’s disease (AD) and other neurodegenerative conditions (Gargouri et al., 2018). Recent studies have demonstrated that GBE was able to reduce neuroinflammatory activation by targeting the COX/PGE2 pathway and mitogen activated protein kinases (MAPKs) pathway (Gargouri et al., 2018). Besides, GBE could scavenge oxidative-free radicals, down-regulate some of the inflammatory mediators involved in the inflammatory responses, including TNF-α, NF-κB p65 and IL-6 (Zhou et al., 2006). However, these researches only displayed the therapeutic outcomes of GBE, but seldom elucidate its underlying pharmacological mechanism. The present study aimed to investigate the potential targets and underlying mechanisms of GBE on neuroinflammation.

NF-κB is an important transcriptional factor in the microglia-mediated inflammatory response in the CNS, and its activation is known to be associated with the translocation of pro-inflammatory cytokines outside the cell in LPS-induced microglial BV-2 cells (Mendonca et al., 2018; Hilliard et al., 2020). Previous studies have shown that amyloid-beta (Aβ) plaques are the distinguishing hallmarks of the AD state, and the plaques could activate NF-κB signaling, followed by the release of critical cytokines related to inflammation. In the present study, the effects of GBE on LPS-stimulated NF-κB activation were investigated. Our results illustrated that GBE clearly inactivated NF-κB by blocking the phosphorylation and degradation of IκB-α and the translocation of p65/p50 dimer. GBE also inhibited the DNA-binding activity of p65 and p50 to COX-2 promoter. These results showed that GBE exerted anti-neuroinflammatory effects through inactivation of the NF-κB pathway.

The abundance of miRNAs specifically expressed within neurons indicates their importance, and scientific evidence suggests that dysregulation of miRNA expression plays a role in the development of neurodegenerative disorders. MiR-146b-5p plays different roles in the progression of different diseases (Sheng et al., 2018), such as AD. Although studies have demonstrated that miR-146b-5p was downregulated in AD patients compared to healthy volunteers (Wu et al., 2020), the underlying mechanisms have not been expounded. Besides, to our best knowledge, few studies have evaluated the association between GBE and miR-146b-5p. In this study, we found that miR-146b-5p expression was lower in patients with AD, and GBE could effectively promote its expression in both BV-2 and U87 cells. From the two miRNA databases, we identified TRAF6 as a candidate target of miR-146b-5p. Furthermore, GBE-induced miR-146b-5p overexpression negatively regulates TRAF6 by directly binding to the 3′-UTR of TRAF6 and inactivating NF-κB pathways, thereby exhibiting anti-neuroinflammatory activity.

## Conclusion

Our data clearly show that GBE exhibits effective anti-neuroinflammatory activity by activating miR-146b-5p expression, which can directly bind to the 3′-UTR of TRAF6 and inhibit its expression. Therefore, GBE attenuates the transcriptional activity of NF-κB and the expression of pro-inflammatory cytokines in microglial cells, such as IL-1β, IL-6, TNF-α, and COX-2. In the present study, we revealed the anti-neuroinflammatory mechanism of GBE and believe that GBE plays a potential role in the prevention of neurodegenerative diseases.

## Data Availability

The original contributions presented in the study are included in the article/Supplementary Materials, further inquiries can be directed to the corresponding authors.
