# Supplementary material for: MiR-146b-5p/TRAF6 axis is essential for Ginkgo biloba L. extract GBE to attenuate LPS-induced neuroinflammation
Source: Front Pharmacol. 2022 Aug 24;13:978587. doi: 10.3389/fphar.2022.978587 (PMC9449131; doi:10.3389/fphar.2022.978587)
Supplement: Supplementary file 1 [file DataSheet1.PDF]

银杏叶提取物注射液
Extract of Ginkgo Biloba Leaves Injection

请仔细阅读银杏叶提取物注射液的作用说明，并在药师指导下购买和使用。

【药品名称】银杏叶提取物注射液

【通用名】银杏叶提取物注射液

【成分】每支含有银杏叶提取物17.5mg，其中银杏黄酮苷4.2mg。 辅料为山梨醇、乙醇、氢氧化钠。

【适应症】主要用于脑部、周围血流循环障碍。 1. 急慢性脑功能不全及其后遗症：脑卒中、注意力不集中、记忆力衰退、痴呆。 2. 耳部血流及神经障碍：耳鸣、眩晕、听力减退、耳迷路综合征。 3. 眼部血流及神经障碍：糖尿病引起的视网膜病变及神经障碍、老年黄斑变性、视力模糊、慢性青光眼。 4. 周围循环障碍：各种周围动脉闭塞症、间歇性跛行症、手脚麻痹冰冷、四肢酸痛。

【包装规格】5ml:17.5mg(含银杏黄酮苷4.2mg)

【用法用量】注射治疗：每天或每隔一天深部肌肉注射或缓慢静脉推注（病人平卧）5毫升本品。 输液治疗：根据病情，通常一日1～2次，一次2～4支。若必要时可调整剂量至一次5支，一日2次。给药时可将本品溶于生理盐水、葡萄糖输液或低分子右旋糖酐或羟乙基淀粉中，混合比例为1：10。若输液为500ml，则静滴速度应控制在大约2～3小时。后续治疗可以口服银杏叶提取物片剂或滴剂。或遵医嘱。

【不良反应】本品耐受性良好，可见胃肠道不适、头痛、血压降低、过敏反应等现象，一般不需要特殊处理即可自行缓解。长期静注时，应改变注射部位以减少静脉炎的发生。

【禁忌】对本品中任一成分过敏者禁用。

【注意事项】1. 银杏叶提取物注射液不影响糖分代谢，因此适用于糖尿病病人。 2. 高乳酸血症（lactacidosis）、甲醇中毒者、果糖山梨醇耐受性不佳者及1， 6－二磷酸果糖酶缺乏者，给药剂量每次不可超过25毫升。 3. 本品不能与其他药物混合使用。 4. 过期不能使用。

【孕妇用药】对妊娠期的使用报告不多，基于安全性考虑，妊娠期不建议使用此药。使用本品期间，如果出现任何不良事件和/或不良反应，请咨询医生。同时使用其他药品，请告知医生。

【儿童用药】尚不明确。

【老年用药】尚不明确。

【药物相互作用】银杏叶提取物注射液应避免与小牛血提取物制剂混合使用。

【药理作用】1.自由基的清除作用：清除机体内过多的自由基，抑制细胞膜的脂质发生过氧化反应，从而保护细胞膜，防止自由基对机体造成的一系列伤害。 2.对循环系统的调整作用：通过刺激儿茶酚胺的释放和抑制降解，以及通过刺激前列环素和内皮舒张因子的生成而产生动脉舒张作用，共同保持动脉和静脉血管的张力。 3.血液动力学改善作用 ：具有降低全血粘稠度，增进红血球和白血球的可塑性，改善血液循环的作用。 4.组织保护作用：增加缺血组织对氧气及葡萄糖的供应量，增加某些神经递质受体的数量，如毒蕈碱样、去甲肾上腺素以及五羟色胺受体。

【批准文号】国药准字H20070226

【生产厂家】悦康药业集团有限公司

Please read carefully the instructions for the action of Ginkgo biloba extract injection, and purchase and use it under the guidance of a pharmacist.

[Drug name] Extract of Ginkgo Biloba Leaves Injection
[Common name] Extract of Ginkgo Biloba Leaves Injection
[Ingredients] Each stick contains 17.5mg of Ginkgo biloba extract, including 4.2mg of ginkgo flavonoid glycosides. The excipients are sorbitol, ethanol and sodium hydroxide.
[Indications] Mainly used for brain and peripheral blood circulation disorders. 1. Acute and chronic brain insufficiency and its sequelae: stroke, inattention, memory loss, dementia. 2. Ear blood flow and nerve disorders: tinnitus, vertigo, hearing loss, ear labyrinth syndrome. 3. Eye blood flow and neurological disorders: diabetes-induced retinopathy and neurological disorders, age-related macular degeneration, blurred vision, chronic glaucoma. 4. Peripheral circulation disorders: various peripheral arterial occlusions, intermittent claudication, numbness and cold hands and feet, and aching limbs.

[Package specification] 5ml: 17.5mg (containing 4.2mg of ginkgo flavone glycosides)

[Usage and Dosage]Injection therapy: 5 ml of this product is injected into the deep muscle or slow intravenous injection (the patient is supine) every day or every other day. Infusion therapy: According to the condition, usually 1 to 2 times a day, 2 to 4 sticks at a time. If necessary, the dose can be adjusted to 5 at a time, 2 times a day. During administration, this product can be dissolved in normal saline, glucose infusion or low molecular weight dextran or hydroxyethyl starch, and the mixing ratio is 1:10. If the infusion is 500ml, the intravenous infusion rate should be controlled within about 2 to 3 hours. Follow-up treatment can be oral ginkgo biloba extract tablets or drops. or as directed by a doctor.

[Adverse reactions] This product is well tolerated, and symptoms such as gastrointestinal discomfort, headache, lower blood pressure, and allergic reactions can be seen. Generally, it can be relieved by itself without special treatment. During long-term intravenous injection, the injection site should be changed to reduce the occurrence of phlebitis.

[Contraindications] Those who are allergic to any of the ingredients in this product are prohibited.

[Notes] 1. Ginkgo biloba extract injection does not affect sugar metabolism, so it is suitable for diabetic patients. 2. For patients with lactic acidosis, methanol poisoning, poor tolerance to fructose sorbitol and 1,6-bisphosphofructase deficiency, the dosage should not exceed 25 ml each time. 3. This product cannot be mixed with other drugs. 4. Expired cannot be used.

[Pregnant women use] There are not many reports on the use of this drug during pregnancy. Based on safety considerations, this drug is not recommended for use during pregnancy. If you experience any adverse events and/or adverse reactions while using this product, please consult your doctor. If you are using other medicines at the same time, please inform your doctor.

[Pediatric Use] Not yet clear.

[Geriatric medication] Not yet clear.

[Drug Interactions] Ginkgo biloba extract injection should avoid mixing with calf blood extract preparations.

[Pharmacological action] 1. The scavenging effect of free radicals: remove excess free radicals in the body, inhibit the lipid peroxidation of cell membranes, thereby protecting the cell membranes and preventing a series of damages caused by free radicals to the body. 2. Regulating effect on the circulatory system: It produces arterial relaxation by stimulating the release of catecholamines and inhibiting degradation, as well as stimulating the production of prostacyclin and endothelial relaxation factor, and jointly maintains the tension of arterial and venous blood vessels. 3. Hemodynamic improvement: It can reduce the viscosity of whole blood, increase the plasticity of red blood cells and white blood cells, and improve blood circulation. 4. Tissue protection: increase the supply of oxygen and glucose to ischemic tissue, and increase the number of certain neurotransmitter receptors, such as muscarinic, norepinephrine and serotonin receptors.

[Approval No.] Guoyao Zhunzi H20070226

[Manufacturer] Yuekang Pharmaceutical Group Co., Ltd.
